# Diagnostic Yield of Genome Sequencing in an Iranian Exome‐Negative Autosomal‐Recessive Intellectual Disability Cohort

**DOI:** 10.1155/humu/4623457

**Published:** 2026-07-01

**Authors:** Ebrahim Shokouhian, Masoumeh Moslemi, Masoumeh Goleyjani Moghadam, Negar Molaei, Parnian Alagha, Azadeh Reshadmanesh, Sanaz Arzhangi, Fatemeh Ghodratpour, Mert Celik, Ilayda Selcen Kadioglu, Masoud Edizadeh, Mohammad Reza Akbari, Kimia Kahrizi, Hossein Najmabadi

**Affiliations:** ^1^ Genetics Research Center, University of Social Welfare and Rehabilitation Sciences, Tehran, Iran, uswr.ac.ir; ^2^ Department of Bioinformatics, Genoks Genetic Diagnosis Center, Ankara, Turkey; ^3^ Women′s College Research Institute, University of Toronto, Toronto, Ontario, Canada, utoronto.ca

**Keywords:** copy number variation, deep intronic, diagnostic yield, genome sequencing, intellectual disability, novel gene

## Abstract

Intellectual disability (ID) affects approximately 1%–3% of the population and spans diverse clinical presentations with marked genetic heterogeneity, especially in consanguineous populations where autosomal‐recessive ID is common. Despite advances in diagnostic methods, ~50% of individuals with ID remain without a molecular diagnosis. Genome sequencing (GS) can detect variant classes poorly captured by other methodologies, including deep intronic splice changes and structural variants. We performed short‐read GS on 38 Iranian autosomal recessive intellectual disability (ARID) families that remained unsolved after exome sequencing (ES) and two phases of reanalysis. Sequencing and variant calling were performed on DRAGEN Bio‐IT Platform with GRCh38 and variants were annotated in Golden helix Varseq software and the AnnotSV tool. GS yielded diagnoses in 2 of 38 families (5.3%), identifying a homozygous deep‐intronic *ATP8A2* variant (c.1473 + 519C > T) that creates a cryptic donor site and a 57‐bp pseudoexon, and a homozygous ~103‐kb *CNTNAP2* deletion removing the in‐frame Exon 2. Additionally, GS uncovered a homozygous *NCOR1* missense variant that represents a novel candidate gene for ARID, but further studies are required to confirm the gene–disease association. The *ATP8A2* and *CNTNAP2* variants were uniquely detectable by GS. In conclusion, in a challenging, ES‐negative ARID cohort, GS provided an additional ~5.3% diagnostic yield by uncovering a noncoding splice alteration and a large intragenic deletion. These results underscore GS as a valuable, though still modest, tool over ES and highlight significant interpretation challenges in noncoding regions. Continued advances in functional assays and complementary long‐read technologies will be essential to further reduce the diagnostic gap in unresolved ID.

## 1. Introduction

Intellectual disability (ID) affects approximately 1%–3% of the population and is highly heterogeneous in genetic etiology [1]. Reportedly 30%–50% of ID is attributable to genetic causes, often involving single‐gene variants [2]. In populations with high consanguinity, autosomal recessive forms of ID (ARID) are common. The heterogeneous genetic diversity and overlap with neurodevelopmental disorders (NDD) pose a major diagnostic challenge [3].

Due to the heterogeneous nature of ID and related NDDs, genome‐wide tests have become first‐line diagnostics. Chromosomal microarray (CMA) traditionally serves as a first‐tier test, but exome sequencing (ES) has emerged as a more powerful diagnostic tool. Studies have shown that ES should be one of the first options for patients with undiagnosed NDD, supported by a diagnostic yield of about 36% (range ~8%–90%). However, even with optimal pipelines and periodic reanalysis, studies report ES diagnostic rates in ID on the order of 25%–46%, leaving roughly 50% of cases undiagnosed after exome testing [4].

Genome sequencing (GS) promises to close part of this diagnostic gap by capturing variant types that ES cannot. Short‐read GS yields an unbiased coverage of the entire genome, including the noncoding and regulatory regions. Crucially, GS can simultaneously detect structural variants (SVs). Advances in bioinformatics and the continued reduction in sequencing costs have increased interest in large‐scale GS studies in rare‐disease cohorts [5]. Indeed, several recent studies have shown that GS can identify additional diagnoses in previously ES‐negative neurodevelopmental disorder cases. Reported yields typically range from 3% to 16% for variant types uniquely detectable by GS, such as deep‐intronic splice‐altering variants and SVs missed by exome‐based methods (Table 1). These results emphasize the added but often limited contribution of GS in revealing genetic causes not captured by ES. Therefore, a significant diagnostic gap remains even with GS, with approximately half of patients with ID still lacking a molecular diagnosis.

**Table 1:** Diagnostic yields from studies using GS on ID/DD related cohorts of patients with negative ES.

Author (reference)	Sample size	Diagnostic yield	GS‐unique yields	Methodology
Álvarez‐Mora et al. [6]	87 families	31/87 families (36%)	1/12 family (8%)	ES was performed on 87 families with NDDs and then GS for 12 families with negative ES.
Sun et al. [7]	100 patients	23/100 patients (23%)	16/100 patients (16%)	GS on 100 pediatric GDD/ID patients with at least one prior genomic diagnostic test.
Hamanaka et al. [8]	260 families	55/260 families (21%)	24/260 families (10.5%)	GS on 260 patients affected by varying degrees of ID/DD with prior negative ES.
Abe‐Hatano et al. [9]	44 families	12/44 families (27%)	All could theoretically be solved by ES	The study used GS as a single step diagnostic tool for 45 patients from 44 families with ID.
Kim et al. [10]	52 patients	10/52 patients (19.2%)	All variants overlap coding regions	Trio GS on 52 patients that had undergone chromosome analysis, CMA, and clinical ES.
Palmer et al. [11]	30 patients	19/30 patients (63.3%)	3/30 patients (10%)	GS on 30 patients with ES negative (15 patients) and multigene panel (15 patients).
Shin et al. [12]	78 patients	26/78 patients (33.3%)	3/78 patients (3.8%)	GS on 78 pediatric patients affected by NDDs with prior negative CMA and/or ES.
Dias et al. [13]	74 patients	51/74 patients (69%)	1/32 patients (3.1%)	GS was performed on 32 ES negative patients from a 74 patients ID cohort. In addition to GS, episignatures and long‐read sequencing were also utilized.

The Genetics Research Center (GRC) at the University of Social Welfare and Rehabilitation Sciences (USWRS), Tehran, Iran, has led a more than a decade‐long effort to identify the genetic causes of autosomal recessive intellectual disability (ARID) in the Iranian population. In 2011, homozygosity mapping and targeted next‐generation sequencing were applied to 136 consanguineous families, identifying mutations in 23 known genes and proposing 50 novel ARID candidates [14]. Subsequently, ES was applied to a cohort of 221 families and reported a diagnostic yield of 51%, including 77 novel candidate gene discoveries. This analysis emphasized the extensive genetic heterogeneity of ARID and revealed the efficacy of high‐throughput DNA sequencing for identifying the causal gene defects in consanguineous families [15].

Most recently, reanalysis of ES data from 159 unresolved ARID families using updated clinical assessments and two modern bioinformatic pipelines resulted in the identification of 19 further diagnoses (11.9%) including six novel candidate genes [16]. These studies demonstrate the cumulative value of long‐term cohort follow‐up and systematic data reanalysis.

In this study, we applied GS to 38 ARID patients who had been extensively evaluated by ES (including two phases of reanalysis) without a diagnosis. We identified two new molecular diagnoses: one patient with a homozygous deep intronic variant creating a cryptic splice site, and one with a 103‐kb homozygous deletion spanning a single exon. Additionally, we identified a homozygous missense variant in *NCOR1* as a novel candidate gene for ID that requires further studies to confirm the gene–disease association. These findings illustrate how GS can uncover diverse classes of hidden mutations and novel candidate genes that eluded prior ES analysis, even while highlighting the remaining gaps in our diagnostic capability.

## 2. Methods and Materials

### 2.1. Study Subjects

This study is a continuation of our previously published work and focuses on a subset of families that remained unsolved after prior ES and subsequent reanalysis. We selected 38 Iranian families from a previously reported cohort of 159 families who underwent ES data reanalysis following an initial negative ES result. The selected families each had more than one individual affected by ID. Each family received a thorough clinical review by a medical geneticist to identify newly affected individuals and update phenotypic descriptions. Pedigrees were reconstructed and information on parental relatedness and likely inheritance models was recorded to inform downstream variant interpretation. Families lost to follow‐up or unable to provide additional samples were excluded from further analysis. Written informed consent was obtained from all participating families and the study protocol received approval from the Ethics Committee of the USWRS.

### 2.2. GS

Genomic DNA libraries were prepared using the TruSeq (Illumina, San Diego, California, United States) library preparation kits and sequenced on an Illumina NovaSeq platform (Illumina, San Diego, California, United States) to a mean genome coverage of approximately 30x. Raw sequencing data were processed on the Illumina DRAGEN Bio‐IT Platform v3.9 [17], which performed read alignment to GRCh38 human reference genome, BAM preprocessing and both short variant (SNV/indel) and copy‐number variant (CNV) detection. Resulting variant calls passing standard quality thresholds were exported for downstream interpretation and annotated using VarSeq software Version 2.6.0 (Golden Helix, Inc., Bozeman, MT, https://www.goldenhelix.com/), and the AnnotSV tool v3.4.4 [18] enabling integration of population frequencies, predicted functional impact, and clinical database annotations to support subsequent filtering and prioritization for SNVs/indels and CNVs, respectively.

### 2.3. Variant Prioritization and Filtering

Variants that passed sequence‐quality filters were initially filtered by population frequency using a 0.01 allele‐frequency cutoff specifically for gnomAD v4.1 (Genome Aggregation Database, https://gnomad.broadinstitute.org/. In addition to allele frequency, we inspected total allele counts and homozygous allele counts from gnomAD v4.1, the 1000 Genomes Project [19] and TOPMed Freeze 8 [20] to help assess the rarity of each variant. Filtered variants were then divided into two main groups: genes/variants with documented phenotypes in OMIM and those without an OMIM phenotype. All variants with existing ClinVar entries were examined carefully regardless of their genomic location (coding or noncoding). For novel coding variants, we applied a stepwise prioritization: (1) Predicted loss‐of‐function variants were prioritized first, (2) variants near canonical splice sites or those with strong splice‐impact predictions were next, (3) in‐frame insertion/deletions that did not localize in a repeat region, and (4) missense changes were evaluated using aggregated dbNSFP v5.1 annotations [21]—including scores from REVEL, MutationTaster, SIFT and PolyPhen—to rank likely deleteriousness. For noncoding calls, intragenic variants and those mapping to candidate cis‐regulatory elements were given higher priority than intergenic changes, with CADD v1.6 scores [22] used to sort and rank noncoding candidates. In addition, potentially causative variants were further assessed using SpliceAI [23], Human Splice Finder (HSF) [24], NNSplice [25], and DeepSea [26] for potential splicing or regulatory effect. Throughout this process, we also considered zygosity, segregation within the pedigree and consistency with the expected inheritance model and patient phenotype. Ultimately, all variants were classified according to the American College of Medical Genetics guideline and subsequent recommendation for noncoding variants [27, 28].

### 2.4. PCR Amplification and Sanger Sequencing for Variant Confirmation and Segregation

To confirm the 103 kb deletion, we employed a breakpoint‐focused strategy: Primer pairs were designed to flank each breakpoint, with one primer binding within the deleted interval and the other outside. Three PCR assays were performed per sample: one for the 5 ^′^ breakpoint, one for the 3 ^′^ breakpoint, and one spanning the entire deletion. This strategy enabled the detection of each junction and the discrimination between homozygous, heterozygous, and wild‐type genotypes. PCR products from homozygous individuals were subjected to Sanger sequencing to validate the breakpoints and the deletion junction sequence.

For the deep intronic variant, segregation was first established on genomic DNA by routine PCR and Sanger sequencing. To evaluate the variant′s effect on splicing, total RNA was extracted from whole blood and reverse‐transcribed into cDNA using conventional reverse‐transcription methods. Primers were designed to span the relevant exons and to discriminate aberrant splice forms, and check for the concordance with the results obtained from Sanger sequencing performed on genomic DNA. Due to the observed partial splicing, the altered bands were gel‐purified prior to Sanger sequencing to determine the precise transcript changes induced by the intronic variant.

The short variant identified in the M8600058 family was validated and cosegregation tested by routine PCR amplification of the target region followed by Sanger sequencing.

All primers were designed with Primer3 (https://primer3.ut.ee/) and Sanger sequencing was performed on an ABI 3130 Sequencer (Life Technologies, Carlsbad, California, United States) and resulting chromatograms were aligned to reference sequences and reviewed using CodonCode Aligner to confirm variant identity and inheritance pattern (primer sequences used in this study are provided in Table S1).

## 3. Results

### 3.1. Cohort Description

This study included 38 Iranian families, each with two or more individuals affected by ID, selected because prior ES analyses had not yielded a definitive molecular diagnosis. As a continuation of our earlier cohort study, all families presented with ID or developmental delay (DD). Nevertheless, the cohort exhibited a range of additional clinical features: 10 families (26.3%) presented with microcephaly; 10 families (26.3%) showed neurological manifestations, including seizures, ataxia, spasticity, autism, weakness, or impaired ambulation; three families (7.9%) had visual problems (including myopia, visual impairment, cataracts, or strabismus); three families (7.9%) showed facial dysmorphism (for example, retrognathia or a long face); two families (5.3%) had short stature or growth delay; one family (2.6%) had hearing loss; and one family (2.6%) was affected by hypothyroidism.

### 3.2. Genetic Diagnoses

GS yielded molecular diagnosis in two of the 38 previously unsolved ARID families (5.3%). These findings highlight the ability of GS to detect variant classes that evade exome‐based workflows, including SVs and deep intronic splice‐altering changes. Both variants cosegregated with the respective phenotypes in accordance with autosomal‐recessive inheritance, and were absent or extremely rare in population databases, supporting their pathogenicity.

GS yielded molecular diagnosis in two of the 38 previously unsolved ARID families (5.3%). These findings highlight the ability of GS to detect variant classes that evade exome‐based workflows, including SVs and deep intronic splice‐altering changes. The two diagnostic variants cosegregated with the respective phenotypes in accordance with autosomal‐recessive inheritance and were absent or extremely rare in population databases, supporting their pathogenicity. In addition, a rare missense variant in the novel candidate gene also cosegregated with the respective ID observed in the family; nevertheless, further in vitro functional studies are necessary to validate this finding.

### 3.3. Homozygous Exon 2 Deletion in CNTNAP2 (Family M9000001)

In family M9000001, GS revealed a homozygous ~103 kb deletion of uncertain significance on Chromosome 7 (chr7:146,706,463–146,809,489) encompassing Exon 2 of CNTNAP2 (NM_014141.6) in three affected individuals. These patients presented with normocephaly, normal height, DD, speech delay, and moderate ID. They were able to walk, had no history of seizures, and no aggression (except for one affected sibling). Additionally, they had normal hearing and vision. This deletion removes the interval extending from Intron 1 through Intron 2 and 111 bps of the coding sequence, creating an in‐frame deletion. *CNTNAP2* is associated with Pitt–Hopkins–like Syndrome 1 (MIM#610042), characterized by seizures, severe ID, profound language impairment, autistic features, and behavioral abnormalities—similar to the clinical features of affected members in this family.

**Figure 1:**
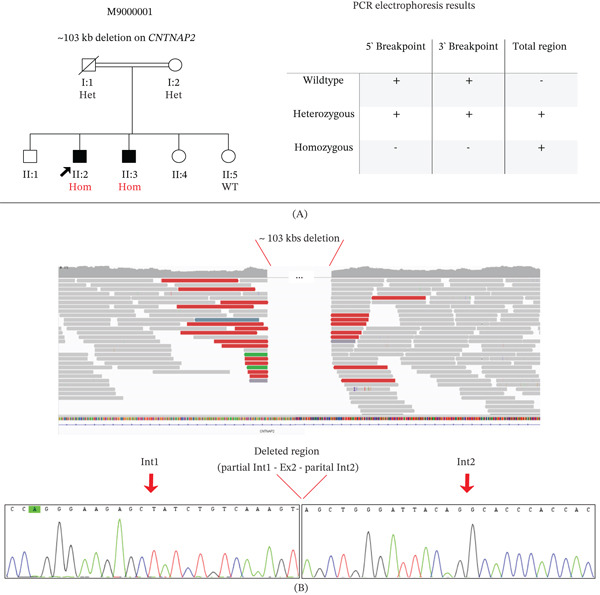
Cosegregation, visualization, and confirmation of the chr7:146,706,463‐146,809,489 deletion spanning Intron 1 to Intron 2, removing exon 2 of the *CNTNAP2* gene. (A) Pedigree and results of PCR electrophoresis. The “+” and “–” symbols indicate the presence or absence of a visible band of the expected size, respectively (the full electrophoresis gel image is provided in Figure S1). (B) Visualization of the 5 ^′^ and 3 ^′^ deletion breakpoints in the Integrative Genomics Viewer (IGV). Sanger sequencing further defined and confirmed the junction sequence at base‐pair resolution.

The deletion was supported by read‐depth and breakpoints evident during visualization (Figure 1). To verify the SV and identify the exact position of breakpoints, a breakpoint‐oriented PCR strategy was implemented. Primers designed to flank each breakpoint generated deletion‐specific PCR products exclusively in homozygous affected individuals, whereas the wild‐type assay failed to amplify. Here, heterozygous carriers also showed amplification of deletion‐specific fragments. In contrast, primer assays targeting the specific 5 ^′^ and 3 ^′^ breakpoints did not amplify in patients with the homozygous deletion, but they produced PCR products in heterozygous individuals, therefore distinguishing homozygous from heterozygous carriers (Figure S1). Sanger sequencing of the breakpoint‐spanning products resolved the junction sequence at base‐pair resolution, confirming the deletion starts at chr7:146,706,463 and ends at chr7:146,809,489 (Figure 1). This deletion was missed entirely by ES and highlights the added diagnostic value of genome‐wide CNV detection.

### 3.4. Homozygous Deep Intronic Variant in ATP8A2 (Family M8600497)

**Figure 2:**
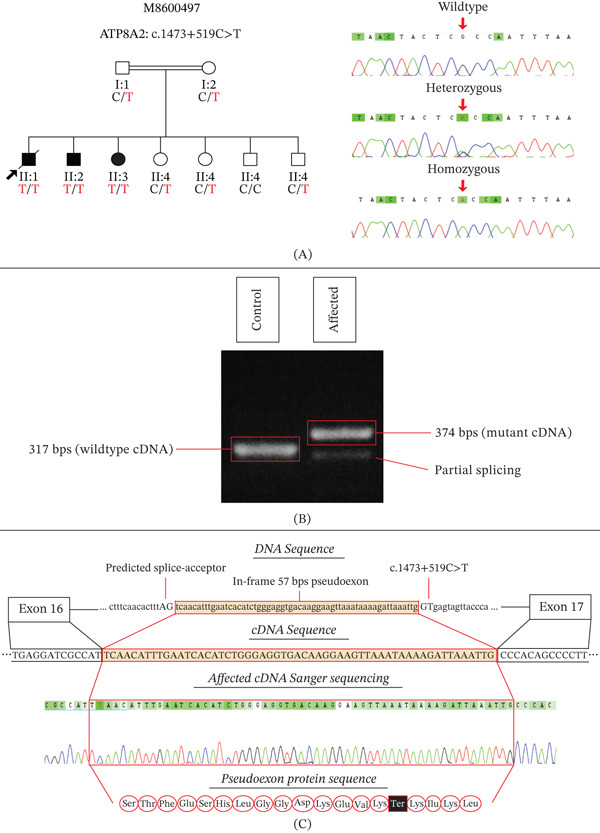
Cosegregation, cDNA analysis and confirmation of the c.1473 + 519C > T deep intronic variant effect on the *ATP8A2* transcript. (A) Pedigree and Sanger sequencing results of the M8600497 family. (B) Electrophoresis results of the *ATP8A2* cDNA PCR products (the full electrophoresis gel image is provided in Figure S2). (C) Deep intronic variant effect on DNA sequence and the assessment and confirmation of the predicted mutant donor and wild type cryptic acceptor splice site using cDNA analysis and Sanger sequencing. Codon phasing revealed the pseudoexon protein sequence showing the inserted premature termination codon.

In family M8600497, whose three individuals presented with normocephaly, normal height, and a history of DD and speech delay. They showed severe ID, seizure disorder, inability to walk, and muscle weakness. Notably, there were no behavioral disturbances, such as aggression or self‐injury. GS identified the homozygous c.1473 + 519C > T substitution in *ATP8A2* (NM_016529.6), located deep within Intron 16 of the gene and it was understandably not captured by previous exome platforms. *ATP8A2* is associated with cerebellar ataxia, impaired intellectual development, and disequilibrium Syndrome 4 (MIM#615268), a phenotype characterized by global DD and congenital cerebellar ataxia. Segregation testing through genomic‐DNA PCR and Sanger sequencing established that the variant was homozygous in affected individuals and heterozygous in parents (Figure 2).

Computational analysis indicated a strong splicing defect: SpliceAI predicted a gain of a cryptic donor site (score 0.57), and HSF similarly suggested formation of a novel splice junction. Interestingly, NNSplice predicted a cryptic AG splice‐acceptor site 57 bp upstream of the newly created donor site at chr13:25,564,491. To determine the functional impact of this intronic change, total RNA extracted from whole blood underwent reverse transcription and targeted cDNA amplification. RT‐PCR demonstrated an aberrant transcript present in affected individuals and heterozygous carriers (Figure S2). Sanger sequencing of gel‐purified products revealed the insertion of an in‐frame 57 bps pseudo‐exon derived from Intron 16 of the *ATP8A2* gene. At the protein level, this is predicted to result in an insertion between p.His491 and p.Pro492 (p.His491_Pro492insSerThrPheGluSerHisLeuGlyGlyAspLysGluValLys∗), which would introduce a premature termination codon within the inserted peptide, presumably leading to truncation of the downstream domains (Figure 2). Based on segregation data, computational predictions, and functional cDNA evidence, this variant was classified as likely pathogenic according to ACMG guidelines (PS3, PM2, PP3, and PP1).

### 3.5. NCOR1 as a Novel Candidate Gene for ARID (Family M8600058)

**Figure 3:**
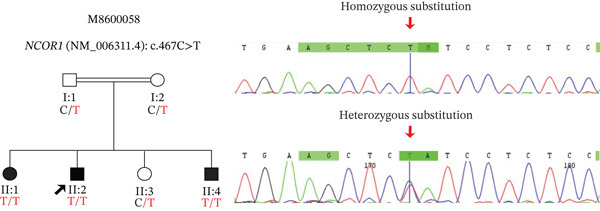
Co‐segregation and results of Sanger sequencing for the M8600058 family.

In family M8600058, GS identified a homozygous missense variant in NCOR1 (NM_006311.4): c.467C > T (p.Pro156Leu), which segregated with disease in an autosomal‐recessive pattern. The affected individuals were born to consanguineous first‐cousin parents. Clinically, all three affected siblings presented with global DD, generalized hypotonia, and cognitive assessment using the Wechsler Intelligence Scale revealed moderate ID (full‐scale IQ scores ranging from 45 to 50). Walking was delayed, achieved between 2 and 4 years of age. Expressive language was severely impaired: two individuals remained nonverbal or did not develop meaningful speech until 12 years of age, with slurred speech in those who gained some expressive ability. Seizures began in early childhood (between 12 months and 6 years of age) and were controlled with antiepileptic medication. Behavioral evaluation noted a short temper in the male siblings. All affected individuals were normocephalic with height appropriate for age, and no major congenital anomalies or significant dysmorphic features were reported. Vision and hearing were normal (Figure 3).

The variant was absent from major population databases (including gnomAD, TOPMed, and Iranome) and was predicted to be deleterious by multiple in silico tools, including CADD (score = 25.4), PROVEAN, VEST4, DEOGEN2, LIST‐S2, and M‐CAP. The altered residue lies in the N‐terminal region of NCOR1 and affects a highly conserved proline, supported by a ConSurf conservation grade of 9, a phyloP 100‐way score of 6.499, and a GERP++ RS score of 5.04, indicating strong evolutionary constraint and functional relevance. Moreover, functional in silico and thermodynamic analyses using I‐Mutant 2.0 [29] and MuPro [30] indicated a destabilizing effect on the protein, but further in vitro analysis is required to confirm these results.

## 4. Discussion

In this cohort of 38 previously ES‐negative ARID families, GS delivered a definitive diagnosis in two cases (5.3%). Each of these two illustrates a distinct class of variant undetected by prior exome analysis. The M8600497 family was identified with the homozygous deep intronic c.1473 + 519C > T variant in the *ATP8A2* gene creating a cryptic donor splice site, and another family carried a homozygous ~103‐kb deletion spanning from Intron 1 to Intron 2 of the *CNTNAP2* gene removing Exon 2. Additionally, a third family harbored the homozygous c.467C > T missense change in the *NCOR1* gene, which represents a novel candidate gene for ID not previously associated with the disorder and requires further validation. Notably, due to the inherent limitations of the ES methodology, the two causal variants were not called during ES data analysis and subsequent reanalysis. The *NCOR1* variant, by contrast, is located in a coding exon and could have been captured by ES—highlighting the importance of periodic ES data reanalysis.

*CNTNAP2* encodes CASPR2, a juxtaparanodal protein in myelinated axons that mediates neuron–glia interactions and organizes voltage‐gated potassium channel complexes, therefore contributing to synaptic homeostasis [31]. Its large extracellular N‐terminus begins with a signal peptide followed by a discoidin (DSC) domain that contributes to ligand recognition, cell–cell/matrix adhesion, and proper trafficking to the neuronal surface [32]. Exon 2 of *CNTNAP2* partially encodes the polypeptide sequence that forms the DSC domain. The homozygous ~103‐kb deletion identified in family M9000001 spans Intron 1 to Intron 2 of *CNTNAP2* and removes Exon 2 (111 bp), resulting in the in‐frame p.Gln33_Gly70del. This alteration is expected to eliminate elements essential for proper DSC folding and to disrupt CASPR2′s ability to bind contactin‐2 and to cluster potassium channels [32], leading to the known *CNTNAP2* related phenotypes.

Biallelic CNVs removing multiple exons of the *CNTNAP2* gene, including Exon 2 (Exons 2–3, 2–8, or 2–9), or compound events with at least one allele overlapping Exon 2, recurrently cause severe neurodevelopmental phenotypes with epilepsy and absent speech [32–34]. By contrast, heterozygous intragenic deletions overlapping Exon 2 have been described with variable expressivity, from unaffected carriers to individuals with isolated language delay, epilepsy or autism spectrum disorder, suggesting dosage sensitivity with incomplete penetrance [32, 35]. Together, these data indicate that Exon 2 is essential for the CASPR2 function.

*ATP8A2* encodes the phospholipid‐transporting ATPase IB that translocates phospholipids, for example, phosphatidylserine, from the outer to the inner layer of cell membranes, leading to the membrane lipid asymmetry in neurons. This protein is highly expressed in the central nervous system, testis, and retina [36]. Biallelic loss‐of‐function variants in *ATP8A2* are known to cause an autosomal recessive neurodevelopmental disorder (CAMRQ4, MIM#615268) characterized by early‐onset cerebellar ataxia, ID, severe hypotonia, and hyperreflexia [37, 38].

In family M8600497, GS revealed the homozygous c.1473 + 519C > T variant in Intron 16 of *ATP8A2,* which prior ES failed to capture due to its noncoding location. This nucleotide substitution was predicted to create a novel splice donor site (SpliceAI, NNSplice, and HSF) and lead to aberrant inclusion of a 57‐base pseudoexon from Intron 16. On cDNA analysis, the mutant transcript contains an in‐frame insertion of 19 codons including a premature stop codon at the Exons 16–17 junction site (p.His491_Pro492ins∗), which is expected to create a truncated protein. The loss of the downstream transmembrane and catalytic domains is predicted to abolish *ATP8A2*′s flippase activity.

According to the best of current knowledge and our research, c.1473 + 519C > T represents the first deep intronic pathogenic variant reported in *ATP8A2*. Previously, the most distal intronic mutation in this gene was a homozygous c.1580–18C > G in Intron 17, which created an alternative acceptor site and caused retention of 17 intronic base pairs with a resultant premature stop codon, leading to mild manifestations of the phenotype, including ataxia, near normal cognition, and mild pyramidal signs [39]. In contrast, the c.1473 + 519C > T variant in Intron 16 identified here is consistent with the severe CAMRQ4 clinical presentation observed in the affected siblings. This case thus underscores how cryptic deep intronic mutations can activate pseudoexon inclusion and disrupt gene function, and highlights the utility of GS in uncovering such noncoding variants in ES‐negative cases.

In addition to the above findings, a homozygous missense variant in NCOR1 (chr17:16165130G > A; c.467C > T (p.Pro156Leu)) in family M8600058 was identified as a novel candidate gene for ARID, resolved primarily through data reanalysis rather than through GS‐specific variant detection. Although NCOR1 has been implicated in neurodevelopmental phenotypes primarily through de novo heterozygous variants (including autism spectrum features) [17], our data suggest a possible recessive disease model in which biallelic disruption may contribute to ID in this consanguineous pedigree, though further studies are needed to confirm this association. The variant segregated with disease under an autosomal‐recessive pattern and was absent from large population databases. Multiple in silico predictors supported deleteriousness (SIFT/PolyPhen‐2/MutationTaster; CADD 26.5), evolutionary constraint analyses indicated that Pro156 is highly conserved (ConSurf grade 9), and thermodynamic analysis using the I‐Mutant 2 and MuPro indicates a destabilizing effect on protein structure, strengthening the likelihood of functional impact but not sufficient to establish causality without additional functional validation.

The Pro156 residue localizes to the N‐terminal intrinsically disordered region of NCOR1, adjacent to a predicted coiled‐coil segment that mediates protein–protein interactions. Although outside canonical repression/SANT domains, the N‐terminus is thought to serve scaffolding and complex‐assembly roles, including recruitment of transcriptional regulators and chromatin‐modifying partners such as HDAC3‐associated repression machinery [40–42]. Substitution of a rigid proline with leucine may alter local conformational properties and impair interaction dynamics, plausibly disturbing neuronal gene regulatory programs. This interpretation is consistent with broader experimental literature linking NCOR1/NCOR2–HDAC3 complexes to neurodevelopmental processes, neuronal/glial cell‐type specificity, and pathways relevant to synaptic plasticity and cognition [43, 44].

Our results show that applying GS to ES‐negative ARID cohort provided an additional ~5.3% diagnostic yield. Studies have noted that short‐read GS provides roughly a 3%–16% absolute increase in diagnostic yield compared with ES [45]. Hamanaka et al. reported a 10.5% GS‐specific yield after excluding cases theoretically detectable by exome [8], whereas Dias et al. found only one truly GS‐unique diagnosis among 32 families (3%) [13]. In a study by Shin et al. on 78 families, 33.3% of patients were diagnosed via GS, but only three cases involved variant types not detectable by ES [12]. These findings suggest that the benefit of GS after prior exome testing is often limited (Table 1).

Our diagnostic yield also shows the challenging nature of our cohort, in which highly consanguineous families had already undergone ES and subsequent two‐phase data reanalysis. Therefore, GS served mainly to uncover structural and noncoding variants missed by earlier methods. As a result, our study further illustrates the interpretive challenges inherent in GS, particularly for noncoding variants. Although short‐read GS captures deep intronic and regulatory regions, functional interpretation remains limited [46]. This challenge is partly due to the marked under‐representation of such variants in public databases and the scientific literature. For example, only 0.34% of high‐confidence pathogenic variants are located within 2 kb upstream of a gene or within untranslated regions. Moreover, these variants are disproportionately classified as variants of uncertain significance (VUS); in ClinVar, 63.4% of all UTR variants are annotated as VUS, compared with 44.2% of coding‐sequence variants [28]. Therefore, further research is still required to enhance functional genomic approaches capable of resolving the pathogenicity of noncoding and regulatory variants.

## 5. Conclusion

Taken together, our results show that GS can yield additional diagnoses in ES negative ARID cases by identifying variant classes not captured by ES. In our cohort, two (~5.3%) additional families were identified with likely causal variants, two of which were detectable only by GS. Although the overall yield remains modest, these findings demonstrate the potential of GS to serve as a top‐tier diagnostic method for ID/DD. Continued improvements in variant interpretation, especially in noncoding regions, as well as emerging technologies like long‐read sequencing, will be essential to further reduce the diagnostic gap in unresolved cases of ID.

## Author Contributions

Ebrahim Shokouhian and Masoumeh Moslemi contributed equally to this work and share first authorship.

## Funding

This study was supported by the University of Social Welfare and Rehabilitation Sciences (10.13039/501100008974, 1402/801/A/6/28595, 3084/T/03, 3049/T/02).

## Conflicts of Interest

The authors declare no conflicts of interest.

## Supporting information


**Supporting Information** Additional supporting information can be found online in the Supporting Information section. The supporting information includes the following: Table S1 contains the primer sequences used for PCR and Sanger sequencing, Figure S1 shows validation of the 103 kb *CNTNAP2* intragenic deletion by gel electrophoresis, and Figure S2 illustrates the effect of the *ATP8A2* deep intronic variant on cDNA by gel electrophoresis.

## Data Availability

The data used in this study are available from the corresponding author upon reasonable request.

## References

[1] Ilyas M. , Mir A. , Efthymiou S. , and Houlden H. , The Genetics of Intellectual disability: advancing technology and gene editing, Advancing Technology and Gene Editing. (2020) 9, 10.12688/f1000research.16315.1. PMC6966773 31984132

[2] Kaufman L. , Ayub M. , and Vincent J. B. , The Genetic Basis of Non-Syndromic Intellectual Disability: A Review, Journal of Neurodevelopmental Disorders. (2010) 2, no. 4, 182–209, 10.1007/s11689-010-9055-2, 21124998. 21124998 PMC2974911

[3] Musante L. and Ropers H. H. , Genetics of Recessive Cognitive Disorders, Trends in Genetics. (2014) 30, no. 1, 32–39, 10.1016/j.tig.2013.09.008. 24176302

[4] Srivastava S. , Love-Nichols J. A. , Dies K. A. , Ledbetter D. H. , Martin C. L. , Chung W. K. , Firth H. V. , Frazier T. , Hansen R. L. , Prock L. , Brunner H. , Hoang N. , Scherer S. W. , Sahin M. , Miller D. T. , and NDD Exome Scoping Review Work Group , Meta-Analysis and Multidisciplinary Consensus Statement: Exome Sequencing Is a First-Tier Clinical Diagnostic Test for Individuals With Neurodevelopmental Disorders, Genetics in Medicine: Official Journal of the American College of Medical Genetics. (2019) 21, no. 11, 2413–2421, 10.1038/s41436-019-0554-6, 31182824. 31182824 PMC6831729

[5] Clark M. M. , Hildreth A. , Batalov S. , Ding Y. , Chowdhury S. , Watkins K. , Ellsworth K. , Camp B. , Kint C. I. , Yacoubian C. , Farnaes L. , Bainbridge M. N. , Beebe C. , Braun J. J. A. , Bray M. , Carroll J. , Cakici J. A. , Caylor S. A. , Clarke C. , Creed M. P. , Friedman J. , Frith A. , Gain R. , Gaughran M. , George S. , Gilmer S. , Gleeson J. , Gore J. , Grunenwald H. , Hovey R. L. , Janes M. L. , Lin K. , McDonagh P. D. , McBride K. , Mulrooney P. , Nahas S. , Oh D. , Oriol A. , Puckett L. , Rady Z. , Reese M. G. , Ryu J. , Salz L. , Sanford E. , Stewart L. , Sweeney N. , Tokita M. , van der Kraan L. , White S. , Wigby K. , Williams B. , Wong T. , Wright M. S. , Yamada C. , Schols P. , Reynders J. , Hall K. , Dimmock D. , Veeraraghavan N. , Defay T. , and Kingsmore S. F. , Diagnosis of Genetic Diseases in Seriously Ill Children by Rapid Whole-Genome Sequencing and Automated Phenotyping and Interpretation, Science Translational Medicine. (2019) 11, no. 489, 10.1126/scitranslmed.aat6177, 31019026. PMC9512059 31019026

[6] Álvarez-Mora M. I. , Sánchez A. , Rodríguez-Revenga L. , Corominas J. , Rabionet R. , Puig S. , and Madrigal I. , Diagnostic Yield of Next-Generation Sequencing in 87 Families With Neurodevelopmental Disorders, Orphanet Journal Of Rare Diseases. (2022) 17, no. 1, 10.1186/s13023-022-02213-z, 35183220. PMC8858550 35183220

[7] Sun Y. , Peng J. , Liang D. , Ye X. , Xu N. , Chen L. , Yan D. , Zhang H. , Xiao B. , Qiu W. , Shen Y. , Pang N. , Liu Y. , Liang C. , Qin Z. , Luo J. , Chen F. , Wang J. , Zhang Z. , Wei H. , du J. , Yan H. , Duan R. , Wang J. , Zhang Y. , Liao S. , Sun K. , Wu L. , and Yu Y. , Genome Sequencing Demonstrates High Diagnostic Yield in Children With Undiagnosed Global Developmental Delay/Intellectual Disability: A Prospective Study, Human Mutation. (2022) 43, no. 5, 568–581, 10.1002/humu.24347, 35143101. 35143101

[8] Hamanaka K. , Fujita A. , Miyatake S. , Misawa K. , Koshimizu E. , Uchiyama Y. , Tsuchida N. , Seyama R. , Sakamoto M. , Iwama K. , Nishimura N. , Utsuno Y. , Fu L. , Takizawa M. , Liang Q. , Itai T. , Saida K. , Ohori S. , Kameyama S. , Fukuda H. , Hayashi Y. , Inoue Y. , Goto T. , Ichikawa K. , Kuki I. , Fukuoka M. , Kim K. , Shiohama T. , Shimoda K. , Otsuka K. , Ueda Y. , Cho K. , Yuge K. , Tachi N. , Yoshida M. , Daida A. , Hirasawa K. , Yanagishita T. , Yamamoto T. , Shirai K. , Mehr T. F. , Fattal-Valevski A. , Lev D. , Yokoyama H. , Iwabuchi E. , Saito Y. , Miura M. , Sugai K. , Ishiyama A. , Sasaki M. , Watanabe Y. , Takanashi J. I. , Kim C. A. , Yokochi K. , Tohyama J. , Mori T. , Izumi Y. , Hasegawa Y. , Okamoto N. , Ikeda T. , Osaka H. , Kawai Y. , Omae Y. , Tokunaga K. , Kato M. , Mizuguchi T. , and Matsumoto N. , Genome Sequencing Provides High Diagnostic Yield and New Etiological Insights for Intellectual Disability and Developmental Delay, NPJ Genomic Medicine. (2025) 10, no. 1, 10.1038/s41525-025-00521-4, 40858643. PMC12381280 40858643

[9] Abe-Hatano C. , Iida A. , Kosugi S. , Momozawa Y. , Terao C. , Ishikawa K. , Okubo M. , Hachiya Y. , Nishida H. , Nakamura K. , Miyata R. , Murakami C. , Takahashi K. , Hoshino K. , Sakamoto H. , Ohta S. , Kubota M. , Takeshita E. , Ishiyama A. , Nakagawa E. , Sasaki M. , Kato M. , Matsumoto N. , Kamatani Y. , Kubo M. , Takahashi Y. , Natsume J. , Inoue K. , and Goto Y. I. , Whole Genome Sequencing of 45 Japanese Patients With Intellectual Disability, American Journal Of Medical Genetics Part A. (2021) 185, no. 5, 1468–1480, 10.1002/ajmg.a.62138, 33624935. 33624935 PMC8247954

[10] Kim J. , Lee J. , Kim M. , and Jang D. H. , Diagnostic Yield of Trio Whole-Genome Sequencing in Children With Undiagnosed Developmental Delay or Congenital Anomaly: A Prospective Cohort Study, Diagnostics. (2024) 14, no. 15, 10.3390/diagnostics14151680, 39125556. PMC11312062 39125556

[11] Palmer E. E. , Sachdev R. , Macintosh R. , Melo U. S. , Mundlos S. , Righetti S. , Kandula T. , Minoche A. E. , Puttick C. , Gayevskiy V. , Hesson L. , Idrisoglu S. , Shoubridge C. , Thai M. H. N. , Davis R. L. , Drew A. P. , Sampaio H. , Andrews P. I. , Lawson J. , Cardamone M. , Mowat D. , Colley A. , Kummerfeld S. , Dinger M. E. , Cowley M. J. , Roscioli T. , Bye A. , and Kirk E. , Diagnostic Yield of Whole Genome Sequencing After Nondiagnostic Exome Sequencing or Gene Panel in Developmental and Epileptic Encephalopathies, Neurology. (2021) 96, no. 13, e1770–e1782, 10.1212/WNL.0000000000011655, 33568551. 33568551

[12] Shin S. , Lee J. , Kim Y. G. , Ha C. , Park J. H. , Kim J. W. , Lee J. , and Jang J. H. , Genetic Diagnosis of Children With Neurodevelopmental Disorders Using Whole Genome Sequencing, Pediatric Neurology. (2023) 149, 44–52, 10.1016/j.pediatrneurol.2023.09.003. 37776660

[13] Dias K. R. , Shrestha R. , Schofield D. , Evans C. A. , O′Heir E. , Zhu Y. , Zhang F. , Standen K. , Weisburd B. , Stenton S. L. , and Sanchis-Juan A. , Narrowing the Diagnostic Gap: Genomes, Episignatures, Long-Read Sequencing, and Health Economic Analyses in an Exome-Negative Intellectual Disability Cohort, Genetics In Medicine: Official Journal Of The American College Of Medical Genetics. (2024) 26, no. 5, 101076, 10.1016/j.gim.2024.101076, 38258669. 38258669 PMC11786952

[14] Najmabadi H. , Hu H. , Garshasbi M. , Zemojtel T. , Abedini S. S. , Chen W. , Hosseini M. , Behjati F. , Haas S. , Jamali P. , Zecha A. , Mohseni M. , Püttmann L. , Vahid L. N. , Jensen C. , Moheb L. A. , Bienek M. , Larti F. , Mueller I. , Weissmann R. , Darvish H. , Wrogemann K. , Hadavi V. , Lipkowitz B. , Esmaeeli-Nieh S. , Wieczorek D. , Kariminejad R. , Firouzabadi S. G. , Cohen M. , Fattahi Z. , Rost I. , Mojahedi F. , Hertzberg C. , Dehghan A. , Rajab A. , Banavandi M. J. S. , Hoffer J. , Falah M. , Musante L. , Kalscheuer V. , Ullmann R. , Kuss A. W. , Tzschach A. , Kahrizi K. , and Ropers H. H. , Deep Sequencing Reveals 50 Novel Genes for Recessive Cognitive Disorders, Nature. (2011) 478, no. 7367, 57–63, 10.1038/nature10423, 21937992. 21937992

[15] Hu H. , Kahrizi K. , Musante L. , Fattahi Z. , Herwig R. , Hosseini M. , Oppitz C. , Abedini S. S. , Suckow V. , Larti F. , Beheshtian M. , Lipkowitz B. , Akhtarkhavari T. , Mehvari S. , Otto S. , Mohseni M. , Arzhangi S. , Jamali P. , Mojahedi F. , Taghdiri M. , Papari E. , Soltani Banavandi M. J. , Akbari S. , Tonekaboni S. H. , Dehghani H. , Ebrahimpour M. R. , Bader I. , Davarnia B. , Cohen M. , Khodaei H. , Albrecht B. , Azimi S. , Zirn B. , Bastami M. , Wieczorek D. , Bahrami G. , Keleman K. , Vahid L. N. , Tzschach A. , Gärtner J. , Gillessen-Kaesbach G. , Varaghchi J. R. , Timmermann B. , Pourfatemi F. , Jankhah A. , Chen W. , Nikuei P. , Kalscheuer V. M. , Oladnabi M. , Wienker T. F. , Ropers H. H. , and Najmabadi H. , Genetics of Intellectual Disability in Consanguineous Families, Molecular Psychiatry. (2019) 24, no. 7, 1027–1039, 10.1038/s41380-017-0012-2. 29302074

[16] Fattahi Z. , Shokouhian E. , Peymani F. , Babanejad M. , Beheshtian M. , Edizadeh M. , Molaei N. , Alagha P. , Ghodratpour F. , Keshavarzi F. , Moghadam M. G. , Arzhangi S. , Kahrizi K. , and Najmabadi H. , Improved Diagnostic Yield in Recessive Intellectual Disability Utilizing Systematic Whole Exome Sequencing Data Reanalysis, Clinical Genetics. (2025) 107, no. 6, 612–619, 10.1111/cge.14692, 39748273. 39748273

[17] Behera S. , Catreux S. , Rossi M. , Truong S. , Huang Z. , Ruehle M. , Visvanath A. , Parnaby G. , Roddey C. , Onuchic V. , Finocchio A. , Cameron D. L. , English A. , Mehtalia S. , Han J. , Mehio R. , and Sedlazeck F. J. , Comprehensive Genome Analysis and Variant Detection at Scale Using DRAGEN, Nature Biotechnology. (2025) 43, no. 7, 1177–1191, 10.1038/s41587-024-02382-1, 39455800. PMC12022141 39455800

[18] Geoffroy V. , Herenger Y. , Kress A. , Stoetzel C. , Piton A. , Dollfus H. , and Muller J. , Annotsv: An Integrated Tool For Structural Variations Annotation, Bioinformatics. (2018) 34, no. 20, 3572–3574, 10.1093/bioinformatics/bty304, 29669011. 29669011

[19] Auton A. , Abecasis G. R. , Altshuler D. M. , Durbin R. M. , Bentley D. R. , Chakravarti A. , Clark A. G. , Donnelly P. , Eichler E. E. , Flicek P. , and Gabriel S. B. , A Global Reference for Human Genetic Variation, Nature. (2015) 526, no. 7571, 68–74, 10.1038/nature15393, 26432245. 26432245 PMC4750478

[20] Taliun D. , Harris D. N. , Kessler M. D. , Carlson J. , Szpiech Z. A. , Torres R. , Taliun S. A. G. , Corvelo A. , Gogarten S. M. , Kang H. M. , Pitsillides A. N. , LeFaive J. , Lee S. B. , Tian X. , Browning B. L. , das S. , Emde A. K. , Clarke W. E. , Loesch D. P. , Shetty A. C. , Blackwell T. W. , Smith A. V. , Wong Q. , Liu X. , Conomos M. P. , Bobo D. M. , Aguet F. , Albert C. , Alonso A. , Ardlie K. G. , Arking D. E. , Aslibekyan S. , Auer P. L. , Barnard J. , Barr R. G. , Barwick L. , Becker L. C. , Beer R. L. , Benjamin E. J. , Bielak L. F. , Blangero J. , Boehnke M. , Bowden D. W. , Brody J. A. , Burchard E. G. , Cade B. E. , Casella J. F. , Chalazan B. , Chasman D. I. , Chen Y. D. I. , Cho M. H. , Choi S. H. , Chung M. K. , Clish C. B. , Correa A. , Curran J. E. , Custer B. , Darbar D. , Daya M. , de Andrade M. , DeMeo D. L. , Dutcher S. K. , Ellinor P. T. , Emery L. S. , Eng C. , Fatkin D. , Fingerlin T. , Forer L. , Fornage M. , Franceschini N. , Fuchsberger C. , Fullerton S. M. , Germer S. , Gladwin M. T. , Gottlieb D. J. , Guo X. , Hall M. E. , He J. , Heard-Costa N. L. , Heckbert S. R. , Irvin M. R. , Johnsen J. M. , Johnson A. D. , Kaplan R. , Kardia S. L. R. , Kelly T. , Kelly S. , Kenny E. E. , Kiel D. P. , Klemmer R. , Konkle B. A. , Kooperberg C. , Köttgen A. , Lange L. A. , Lasky-Su J. , Levy D. , Lin X. , Lin K. H. , Liu C. , Loos R. J. F. , Garman L. , Gerszten R. , Lubitz S. A. , Lunetta K. L. , Mak A. C. Y. , Manichaikul A. , Manning A. K. , Mathias R. A. , McManus D. D. , McGarvey S. T. , Meigs J. B. , Meyers D. A. , Mikulla J. L. , Minear M. A. , Mitchell B. D. , Mohanty S. , Montasser M. E. , Montgomery C. , Morrison A. C. , Murabito J. M. , Natale A. , Natarajan P. , Nelson S. C. , North K. E. , O’Connell J. R. , Palmer N. D. , Pankratz N. , Peloso G. M. , Peyser P. A. , Pleiness J. , Post W. S. , Psaty B. M. , Rao D. C. , Redline S. , Reiner A. P. , Roden D. , Rotter J. I. , Ruczinski I. , Sarnowski C. , Schoenherr S. , Schwartz D. A. , Seo J. S. , Seshadri S. , Sheehan V. A. , Sheu W. H. , Shoemaker M. B. , Smith N. L. , Smith J. A. , Sotoodehnia N. , Stilp A. M. , Tang W. , Taylor K. D. , Telen M. , Thornton T. A. , Tracy R. P. , van den Berg D. J. , Vasan R. S. , Viaud-Martinez K. A. , Vrieze S. , Weeks D. E. , Weir B. S. , Weiss S. T. , Weng L. C. , Willer C. J. , Zhang Y. , Zhao X. , Arnett D. K. , Ashley-Koch A. E. , Barnes K. C. , Boerwinkle E. , Gabriel S. , Gibbs R. , Rice K. M. , Rich S. S. , Silverman E. K. , Qasba P. , Gan W. , NHLBI Trans-Omics for Precision Medicine (TOPMed) Consortium , Abe N. , Almasy L. , Ament S. , Anderson P. , Anugu P. , Applebaum-Bowden D. , Assimes T. , Avramopoulos D. , Barron-Casella E. , Beaty T. , Beck G. , Becker D. , Beitelshees A. , Benos T. , Bezerra M. , Bis J. , Bowler R. , Broeckel U. , Broome J. , Bunting K. , Bustamante C. , Buth E. , Cardwell J. , Carey V. , Carty C. , Casaburi R. , Castaldi P. , Chaffin M. , Chang C. , Chang Y. C. , Chavan S. , Chen B. J. , Chen W. M. , Chuang L. M. , Chung R. H. , Comhair S. , Cornell E. , Crandall C. , Crapo J. , Curtis J. , Damcott C. , David S. , Davis C. , Fuentes L. , DeBaun M. , Deka R. , Devine S. , Duan Q. , Duggirala R. , Durda J. P. , Eaton C. , Ekunwe L. , el Boueiz A. , Erzurum S. , Farber C. , Flickinger M. , Fornage M. , Frazar C. , Fu M. , Fulton L. , Gao S. , Gao Y. , Gass M. , Gelb B. , Geng X. P. , Geraci M. , Ghosh A. , Gignoux C. , Glahn D. , Gong D. W. , Goring H. , Graw S. , Grine D. , Gu C. C. , Guan Y. , Gupta N. , Haessler J. , Hawley N. L. , Heavner B. , Herrington D. , Hersh C. , Hidalgo B. , Hixson J. , Hobbs B. , Hokanson J. , Hong E. , Hoth K. , Hsiung C. A. , Hung Y. J. , Huston H. , Hwu C. M. , Jackson R. , Jain D. , Jhun M. A. , Johnson C. , Johnston R. , Jones K. , Kathiresan S. , Khan A. , Kim W. , Kinney G. , Kramer H. , Lange C. , Lange E. , Lange L. , Laurie C. , LeBoff M. , Lee J. , Lee S. S. , Lee W. J. , Levine D. , Lewis J. , Li X. , Li Y. , Lin H. , Lin H. , Lin K. H. , Liu S. , Liu Y. , Liu Y. , Luo J. , Mahaney M. , Make B. , Manson J. A. , Margolin L. , Martin L. , Mathai S. , May S. , McArdle P. , McDonald M. L. , McFarland S. , McGoldrick D. , McHugh C. , Mei H. , Mestroni L. , Min N. , Minster R. L. , Moll M. , Moscati A. , Musani S. , Mwasongwe S. , Mychaleckyj J. C. , Nadkarni G. , Naik R. , Naseri T. , Nekhai S. , Neltner B. , Ochs-Balcom H. , Paik D. , Pankow J. , Parsa A. , Peralta J. M. , Perez M. , Perry J. , Peters U. , Phillips L. S. , Pollin T. , Becker J. P. , Boorgula M. P. , Preuss M. , Qiao D. , Qin Z. , Rafaels N. , Raffield L. , Rasmussen-Torvik L. , Ratan A. , Reed R. , Regan E. , Reupena M.‘. S. , Roselli C. , Russell P. , Ruuska S. , Ryan K. , Sabino E. C. , Saleheen D. , Salimi S. , Salzberg S. , Sandow K. , Sankaran V. G. , Scheller C. , Schmidt E. , Schwander K. , Sciurba F. , Seidman C. , Seidman J. , Sherman S. L. , Shetty A. , Sheu W. H. H. , Silver B. , Smith J. , Smith T. , Smoller S. , Snively B. , Snyder M. , Sofer T. , Storm G. , Streeten E. , Sung Y. J. , Sylvia J. , Szpiro A. , Sztalryd C. , Tang H. , Taub M. , Taylor M. , Taylor S. , Threlkeld M. , Tinker L. , Tirschwell D. , Tishkoff S. , Tiwari H. , Tong C. , Tsai M. , Vaidya D. , VandeHaar P. , Walker T. , Wallace R. , Walts A. , Wang F. F. , Wang H. , Watson K. , Wessel J. , Williams K. , Williams L. K. , Wilson C. , Wu J. , Xu H. , Yanek L. , Yang I. , Yang R. , Zaghloul N. , Zekavat M. , Zhao S. X. , Zhao W. , Zhi D. , Zhou X. , Zhu X. , Papanicolaou G. J. , Nickerson D. A. , Browning S. R. , Zody M. C. , Zöllner S. , Wilson J. G. , Cupples L. A. , Laurie C. C. , Jaquish C. E. , Hernandez R. D. , O’Connor T. D. , and Abecasis G. R. , Sequencing of 53,831 Diverse Genomes From the NHLBI TOPMed Program, Nature. (2021) 590, no. 7845, 290–299, 10.1038/s41586-021-03205-y. 33568819 PMC7875770

[21] Liu X. , Li C. , Mou C. , Dong Y. , and Tu Y. , dbNSFP v4: A Comprehensive Database of Transcript-Specific Functional Predictions and Annotations for Human Nonsynonymous and Splice-Site SNVs, Genome Medicine. (2020) 12, no. 1, 10.1186/s13073-020-00803-9, 33261662. PMC7709417 33261662

[22] Rentzsch P. , Witten D. , Cooper G. M. , Shendure J. , and Kircher M. , CADD: Predicting the Deleteriousness of Variants Throughout the Human Genome, Nucleic Acids Research. (2019) 47, no. 1, D886–D894, 10.1093/nar/gky1016, 30371827. 30371827 PMC6323892

[23] Jaganathan K. , Kyriazopoulou Panagiotopoulou S. , McRae J. F. , Darbandi S. F. , Knowles D. , Li Y. I. , Kosmicki J. A. , Arbelaez J. , Cui W. , Schwartz G. B. , Chow E. D. , Kanterakis E. , Gao H. , Kia A. , Batzoglou S. , Sanders S. J. , and Farh K. K. H. , Predicting Splicing From Primary Sequence With Deep Learning, Cell. (2019) 176, no. 3, 535–548, 10.1016/j.cell.2018.12.015, 30661751. 30661751

[24] Desmet F. O. , Hamroun D. , Lalande M. , Collod-Béroud G. , Claustres M. , and Béroud C. , Human Splicing Finder: An Online Bioinformatics Tool to Predict Splicing Signals, Nucleic Acids Research. (2009) 37, no. 9, 10.1093/nar/gkp215, 19339519. PMC2685110 19339519

[25] Reese M. G. , Eeckman F. H. , Kulp D. , and Haussler D. , Improved Splice Site Detection in Genie, Journal of Computational Biology: A Journal of Computational Molecular Cell Biology. (1997) 4, no. 3, 311–323, 10.1089/cmb.1997.4.311, 9278062. 9278062

[26] Zhou J. and Troyanskaya O. G. , Predicting Effects of Noncoding Variants With Deep Learning-Based Sequence Model, Nature Methods. (2015) 12, no. 10, 931–934, 10.1038/nmeth.3547, 26301843. 26301843 PMC4768299

[27] Richards S. , Aziz N. , Bale S. , Bick D. , Das S. , Gastier-Foster J. , Grody W. W. , Hegde M. , Lyon E. , Spector E. , and Voelkerding K. , Standards and Guidelines for the Interpretation of Sequence Variants: A Joint Consensus Recommendation of the American College of Medical Genetics and Genomics and the Association for Molecular Pathology, Genetics in medicine: official journal of the American College of Medical Genetics. (2015) 17, no. 5, 405–424, 10.1038/gim.2015.30, 25741868. 25741868 PMC4544753

[28] Ellingford J. M. , Ahn J. W. , Bagnall R. D. , Baralle D. , Barton S. , Campbell C. , Downes K. , Ellard S. , Duff-Farrier C. , FitzPatrick D. R. , Greally J. M. , Ingles J. , Krishnan N. , Lord J. , Martin H. C. , Newman W. G. , O’Donnell-Luria A. , Ramsden S. C. , Rehm H. L. , Richardson E. , Singer-Berk M. , Taylor J. C. , Williams M. , Wood J. C. , Wright C. F. , Harrison S. M. , and Whiffin N. , Recommendations for Clinical Interpretation of Variants Found in Non-Coding Regions of the Genome, Genome Medicine. (2022) 14, no. 1, 10.1186/s13073-022-01073-3, 35850704. PMC9295495 35850704

[29] Capriotti E. , Fariselli P. , and Casadio R. , I-Mutant2.0: Predicting Stability Changes Upon Mutation From the Protein Sequence or Structure, Nucleic Acids Research. (2005) 33, W306–W310, 10.1093/nar/gki375, 15980478. 15980478 PMC1160136

[30] Cheng J. , Randall A. , and Baldi P. , Prediction of Protein Stability Changes for Single-Site Mutations Using Support Vector Machines, Proteins. (2006) 62, no. 4, 1125–1132, 10.1002/prot.20810, 16372356. 16372356

[31] Saint-Martin M. , Joubert B. , Pellier-Monnin V. , Pascual O. , Noraz N. , and Honnorat J. , Contactin-Associated Protein-Like 2, a Protein of the Neurexin Family Involved in Several Human Diseases, European Journal Of Neuroscience. (2018) 48, no. 3, 1906–1923, 10.1111/ejn.14081, 30028556. 30028556

[32] St George-Hyslop F. , Kivisild T. , and Livesey F. J. , The Role of Contactin-Associated Protein-Like 2 in Neurodevelopmental Disease and Human Cerebral Cortex Evolution, Frontiers In Molecular Neuroscience. (2022) 15, 1017144, 10.3389/fnmol.2022.1017144, 36340692. 36340692 PMC9630569

[33] Zweier C. , de Jong E. K. , Zweier M. , Orrico A. , Ousager L. B. , Collins A. L. , Bijlsma E. K. , Oortveld M. A. W. , Ekici A. B. , Reis A. , Schenck A. , and Rauch A. , CNTNAP2 and NRXN1 Are Mutated in Autosomal-Recessive Pitt-Hopkins-Like Mental Retardation and Determine the Level of a Common Synaptic Protein in Drosophila, American Journal Of Human Genetics. (2009) 85, no. 5, 655–666, 10.1016/j.ajhg.2009.10.004, 19896112. 19896112 PMC2775834

[34] Rodenas-Cuadrado P. , Pietrafusa N. , Francavilla T. , La Neve A. , Striano P. , and Vernes S. C. , Characterisation of CASPR2 Deficiency Disorder--A Syndrome Involving Autism, Epilepsy and Language Impairment, BMC Medical Genetics. (2016) 17, no. 1, 10.1186/s12881-016-0272-8, 26843181. PMC4739328 26843181

[35] Al-Murrani A. , Ashton F. , Aftimos S. , George A. M. , and Love D. R. , Amino-Terminal Microdeletion Within theCNTNAP2Gene Associated With Variable Expressivity of Speech Delay, Case Reports In Genetics. (2012) 2012, 172408, 10.1155/2012/172408. 23074684 PMC3447220

[36] Chalat M. , Moleschi K. , and Molday R. S. , C-Terminus of the P4-ATPase ATP8A2 Functions in Protein Folding and Regulation of Phospholipid Flippase Activity, Molecular Biology Of The Cell. (2017) 28, no. 3, 452–462, 10.1091/mbc.e16-06-0453, 27932490. 27932490 PMC5341728

[37] Gulsuner S. , Tekinay A. B. , Doerschner K. , Boyaci H. , Bilguvar K. , Unal H. , Ors A. , Onat O. E. , Atalar E. , Basak A. N. , Topaloglu H. , Kansu T. , Tan M. , Tan U. , Gunel M. , and Ozcelik T. , Homozygosity Mapping and Targeted Genomic Sequencing Reveal the Gene Responsible for Cerebellar Hypoplasia and Quadrupedal Locomotion in a Consanguineous Kindred, Genome Research. (2011) 21, no. 12, 1995–2003, 10.1101/gr.126110.111, 21885617. 21885617 PMC3227090

[38] Onat O. E. , Gulsuner S. , Bilguvar K. , Nazli Basak A. , Topaloglu H. , Tan M. , Tan U. , Gunel M. , and Ozcelik T. , Missense Mutation in the ATPase, Aminophospholipid Transporter Protein ATP8A2 Is Associated With Cerebellar Atrophy and Quadrupedal Locomotion, European Journal Of Human Genetics. (2013) 21, no. 3, 281–285, 10.1038/ejhg.2012.170, 22892528. 22892528 PMC3573203

[39] Damásio J. , Santos D. , Morais S. , Brás J. , Guerreiro R. , Sardoeira A. , Cavaco S. , Carrilho I. , Barbot C. , Barros J. , and Sequeiros J. , Congenital Ataxia due to Novel Variant inATP8A2, Clinical Genetics. (2021) 100, no. 1, 79–83, 10.1111/cge.13954. 33682124

[40] Iemolo A. , Montilla-Perez P. , Lai I. C. , Meng Y. , Nolan S. , Wen J. , Rusu I. , Dulcis D. , and Telese F. , A Cell Type-Specific Expression Map of NCoR1 and SMRT Transcriptional Co-Repressors in the Mouse Brain, Journal Of Comparative Neurology. (2020) 528, no. 13, 2218–2238, 10.1002/cne.24886, 32072640. 32072640 PMC7368833

[41] Geiger M. A. , Guillaumon A. T. , Paneni F. , Matter C. M. , and Stein S. , Role of the Nuclear Receptor Corepressor 1 (NCOR1) in Atherosclerosis and Associated Immunometabolic Diseases, Frontiers In Immunology. (2020) 11, 569358, 10.3389/fimmu.2020.569358, 33117357. 33117357 PMC7578257

[42] Sun Z. and Xu Y. , Nuclear Receptor Coactivators (NCOAs) and Corepressors (NCORs) in the Brain, Endocrinology. (2020) 161, no. 8, bqaa083, 10.1210/endocr/bqaa083, 32449767. 32449767 PMC7351129

[43] Cheng K. M. , Hsu W. L. , Ma Y. L. , Liu Y. C. , and Lee E. H. Y. , Novel Role of NCoR1 in Impairing Spatial Memory Through the Mediation of a Novel Interacting Protein DEC2, Cellular And Molecular Life Sciences. (2024) 81, no. 1, 10.1007/s00018-024-05321-0, 38900294. PMC11335199 38900294

[44] Zhou W. , He Y. , Rehman A. U. , Kong Y. , Hong S. , Ding G. , Yalamanchili H. K. , Wan Y. W. , Paul B. , Wang C. , and Gong Y. , Loss of Function of NCOR1 and NCOR2 Impairs Memory Through a Novel GABAergic Hypothalamus-CA3 Projection, Nature Neuroscience. (2019) 22, no. 2, 205–217, 10.1038/s41593-018-0311-1, 30664766. 30664766 PMC6361549

[45] Smirnov D. , Konstantinovskiy N. , and Prokisch H. , Integrative Omics Approaches to Advance Rare Disease Diagnostics, Journal of Inherited Metabolic Disease. (2023) 46, no. 5, 824–838, 10.1002/jimd.12663. 37553850

[46] Awaya T. , Kurosawa R. , and Hagiwara M. , Genome-Wide Functional Annotation and Interpretation of Splicing Variants: Toward RNA-Targeted Therapies, Journal Of Human Genetics. (2025) 1–10, 10.1038/s10038-025-01424-z, 41188449. 41188449

