# Supplementary material for: Diagnostic Yield of Genome Sequencing in an Iranian Exome‐Negative Autosomal‐Recessive Intellectual Disability Cohort
Source: Hum Mutat. 2026 Jul 1;2026:4623457. doi: 10.1155/humu/4623457 (PMC13319903; doi:10.1155/humu/4623457)
Supplement: Supplementary file 1 — Supporting Information Additional supporting information can be found online in the Supporting Information section. The supporting information includes the following: Table S1 contains the primer sequences used for PCR and Sanger sequencing, Figure S1 shows validation of the 103 kb CNTNAP2 intragenic deletion by gel electrophoresis, and Figure S2 illustrates the effect of the ATP8A2 deep intronic variant on cDNA by gel electrophoresis. [file HUMU-2026-4623457-s001.pdf]

Supplementary Table 1. Primer sequences used for PCR and Sanger sequencing.

Primer Name	Primer Sequence	Product size
**ATP8A2-cDNA-F**	CGGAGTAACCTATGGTCACTT	317 bps (Wildtype)<br>374 bps (Mutant)
**ATP8A2-cDNA-R**	ATGATGACTGAGAATGGTGTTC	
**ATP8A2-genomic-F**	CATGGGAAAAGTCTCGAC	506 bps
**ATP8A2-gneomic-R**	GAAGTGGTGTGATGTCGTAG	
**CNTNAP2-START-F**	TGGTAGGGAAATGGAGATGA	782 bps
**CNTNAP2-START-R**	TTCAAATAGTAGAGTCCAGTGCC	
**CNTNAP2-END-F**	TTCTCTACAGCCTCCCAAAC	839 bps
**CNTNAP2-END-R**	TGGCCCTCGTCTTATACCA	
**NCOR1-F**	AGGTGAGAATTTGCATGTATG	671 bps
**NCOR1-R**	AAGCACTGCCATTCCTG	

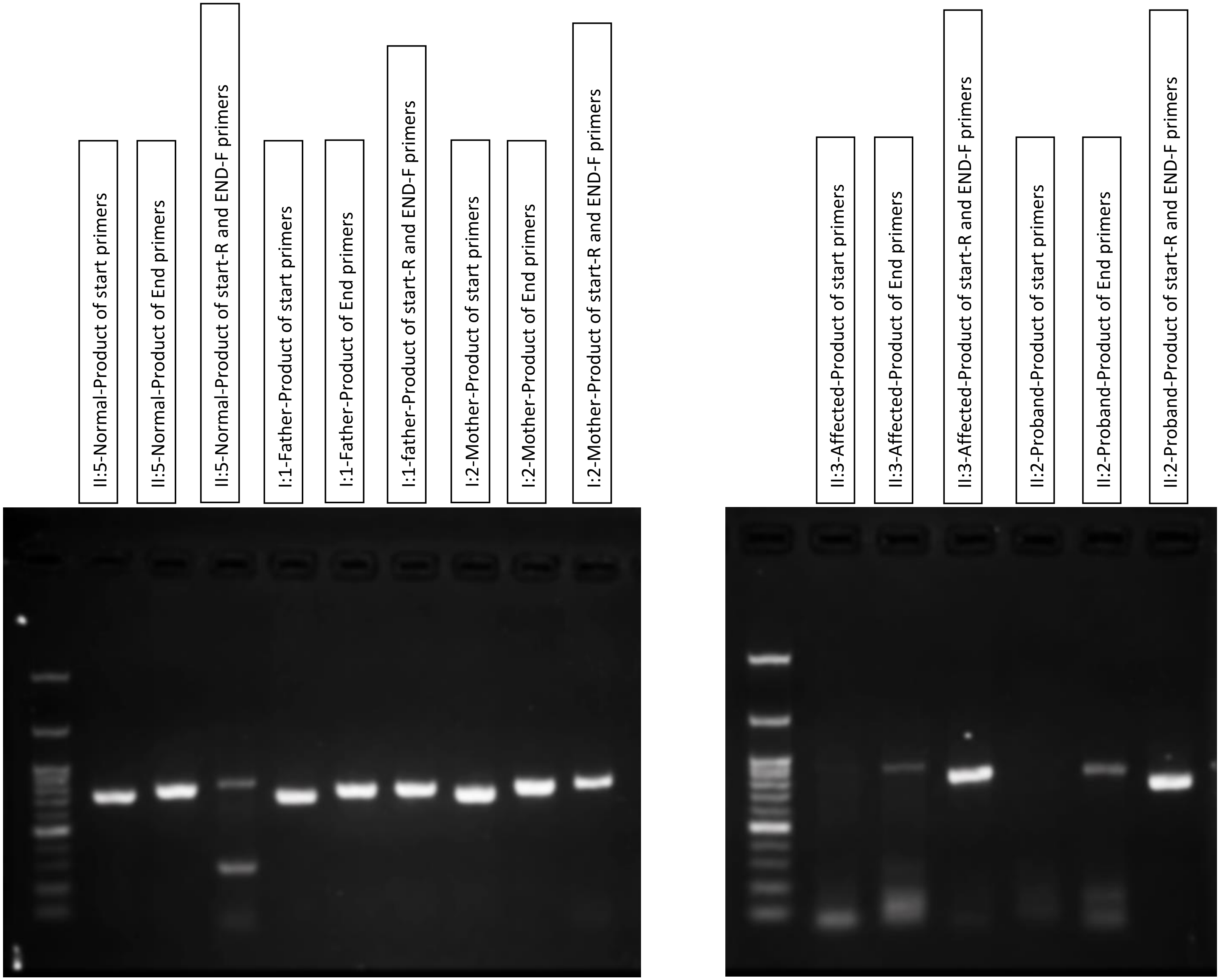

Supplementary Figure 1. Gel Electrophoresis Confirming the 103 kb intragenic Deletion in *CNTNAP2*.

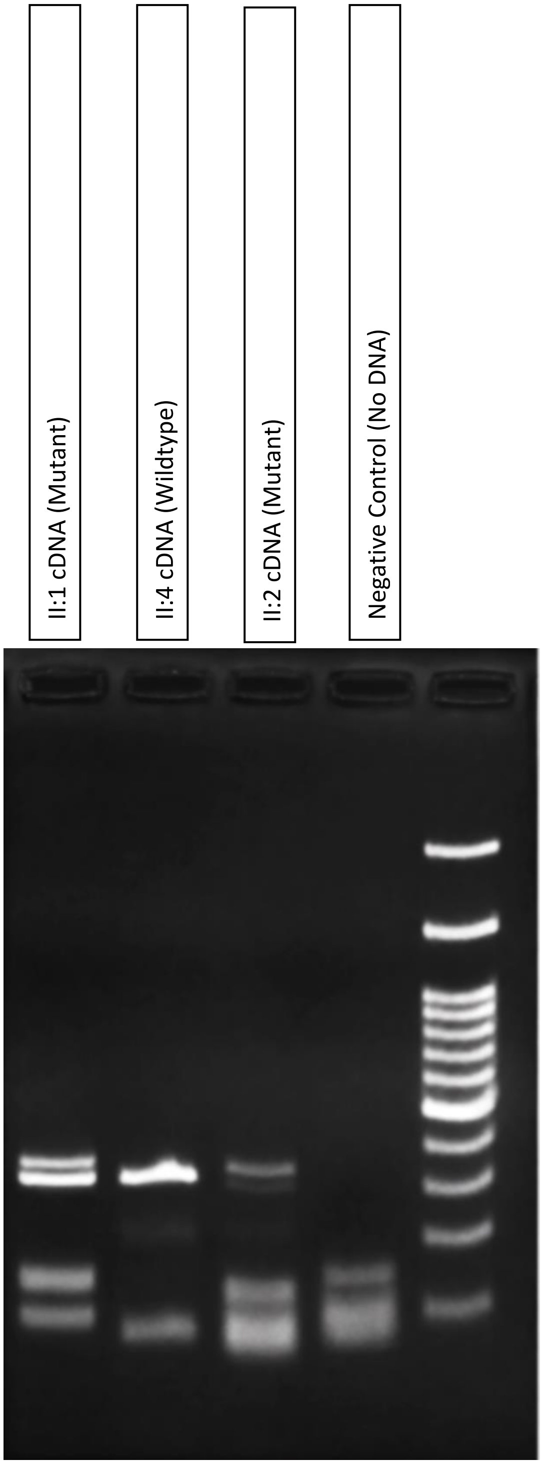

Supplementary Figure 2. Gel electrophoresis of *ATP8A2* cDNA products revealing the impact of the deep intronic variant.
